# Development and clinical application of anti-HER2 monoclonal and bispecific antibodies for cancer treatment

**DOI:** 10.1186/s40164-017-0091-4

**Published:** 2017-11-28

**Authors:** Shengnan Yu, Qian Liu, Xinwei Han, Shuang Qin, Weiheng Zhao, Anping Li, Kongming Wu

**Affiliations:** 1 Department of Oncology, Tongji Hospital of Tongji Medical College, Huazhong University of Science and Technology, 1095 Jiefang Avenue, Wuhan, 430030 China; 2 Department of Interventional Radiology, First Affiliated Hospital of Zhengzhou University, Zhengzhou, 450052 China

**Keywords:** HER2, Trastuzumab, Pertuzumab, T-DM1, Bispecific antibody, Ertumaxomab, MM-111, HER2Bi-aATCs

## Abstract

HER2-targeted immunotherapy consists of monoclonal antibodies (e.g. trastuzumab, pertuzumab), bispecific antibodies (e.g. MM-111, ertumaxomab) and activated T cells armed with anti-HER2 bispecific antibody (HER2Bi-aATC). Trastuzumab is a classic drug for the treatment of HER2 positive metastatic breast cancer. The combined application of pertuzumab, trastuzumab and paclitaxel has been suggested as a standard therapy for HER2 positive advanced breast cancer. The resistance to anti-HER2 antibody has resulted in disease progression. HER2-directed bispecific antibody may be a promising therapeutic approach for these patients. Ertumaxomab enhanced the interaction of immune effector cells and tumor cells. MM-111 simultaneously binds to HER2 and HER3 and blocks downstream signaling. Besides, HER2Bi-aATC is also an alternative therapeutic approach for HER2 positive cancers. In this review, we summarized the recent advancement of HER2-targeted monoclonal antibodies (trastuzumab, pertuzumab and T-DM1) and bispecific antibodies (MM-111, ertumaxomab and HER2Bi-aATC), especially focus on clinical trial results.

## Background

Human epithelial growth factor receptor 2 (HER2) belongs to the receptor tyrosine kinase family, which consists of four members: HER1 (also known as EGFR), HER2 (also known as Neu), HER3 and HER4 [1]. HER2 is a 185-kDa transmembrane glycoprotein containing three components: an extracellular ligand binding domain, a transmembrane domain, and an intracellular domain that has tyrosine kinase activity [2]. Amplification of the *HER2* gene or overexpression of HER2 receptor plays a crucial role in the cellular transformation, carcinogenesis and prognosis of many cancer types [3]. HER2-positive tumors account for about 20–30% breast cancer [4], 20% advanced gastric or gastric or gastro-esophageal junction cancers [5], 5–15% bladder cancers [6], 5–15% cervix cancers [7], 12–15% gallbladder cancers [8], 8–35% endometrium cancers [9], 6–7% ovarian cancers [10], and 15–37% salivary duct cancers [11]. Because of this, detection of the expression level of HER2 is conventional and helpful for doctors to diagnose, especially in patients with breast cancer. In addition to evaluate the expression level of HER2 in primary site by immunohistochemistry staining (IHC) or fluorescence in situ hybridization (FISH), detection of circulating tumor cells (CTCs) is also regarded as a promising method [12]. HER2 is considered as an ideal target for antitumor treatment [13, 14]. Unlike other members, HER2 has no any known natural ligand to bind. It exhibits functions through EGFR-HER2 heterodimers, HER2-HER3 heterodimers, and HER2-HER2 homodimers [15, 16].

**Fig. 1:**
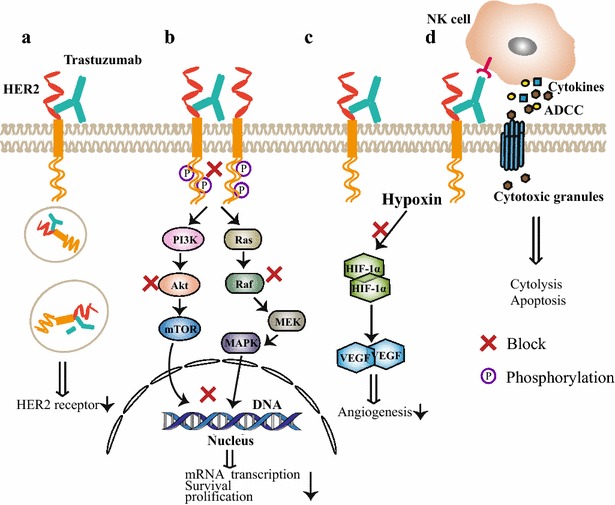
The antitumor mechanisms of anti-HER2 monoclonal antibody (taking an example of trastuzumab). **a** Trastuzumab downregulates HER2 expression by inducing receptor internalization and degradation. **b** Trastuzumab binding to extracellular subdomain IV of HER2 inhibits the homodimerization of HER2 and blocks downstream PI3K/Akt and Ras/Raf/MAPK pathways. **c** Trastuzumab plays a role in inhibiting angiogenesis. **d** Fc fragment of trastuzumab binding to NK cells triggers potent ADCC and secretion of cytokines to kill tumor cell

Until now, several HER2-directed therapies have been approved for the HER2-positive breast cancer and non-small cell lung cancer, including trastuzumab, pertuzumab, T-DM1, lapatinib and afatinib (tyrosine kinase inhibitors which blocked EGFR and HER2) [3, 17]. Trastuzumab, as a classical anti-HER2 antibody, blocked homodimerization of HER2 through binding to the domain IV of HER2 [18]. As to pertuzumab, it can prevent the formation of heterodimerization via binding to HER2 subdomain II [19]. Because of the distinct but complementary modes of action, combination of the two agents could obviously strengthen the blockage of downstream signaling, including phosphoinositide 3-kinase/protein kinase B/mammalian target of rapamycin (PI3K/Akt/mTOR) and Ras/Raf/mitogen-activated protein kinase (MAPK) [20, 21]. Besides, anti-HER2 monoclonal antibodies could increase endocytosis of HER2 receptor, suppress angiogenesis [22, 23], and induce tumor cell lysis through antibody-dependent cell-mediated cytotoxicity (ADCC) [18] (Fig. 1). Ado-trastuzumab emtansine (T-DM1) is an approved antibody drug conjugate for HER2-positive breast cancer. In addition to having the function of trastuzumab, T-DM1 could release the microtubule-inhibitory agent (DM1) after internalization of HER2/T-DM1 complex [3]. Besides, synergistic antitumor functions of HER2 antibody with other antitumor agents have been observed in both in vitro and in vivo studies [24, 25]. However, about 70% patients are resistant to trastuzumab, and some exhibited primary resistance [26, 27]. Aimed at the obstacle, researchers have proposed several corresponding strategies: maintaining trastuzumab therapy after progression [28, 29], combining HER2 inhibitors [30, 31], and developing novel anti-HER2 monoclonal antibodies [32]. Bispecific antibodies, such as blinatumomab, have achieved great success in hematological malignancies [33]. Among those, HER2-targeted bispecific antibodies which introduced to be widely investigated are also regarded as a remarkable solution [34].

**Fig. 2:**
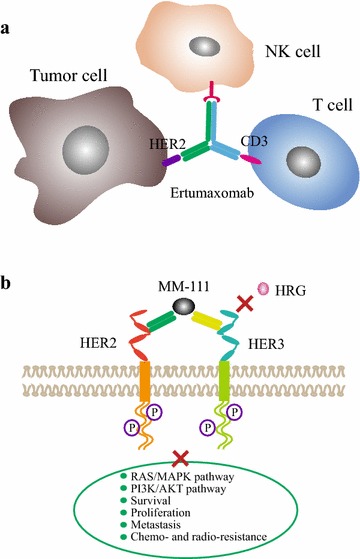
The antitumor mechanisms of HER2-targeted bispecific antibody (taking examples of ertumaxomab and MM-111). **a** Ertumaxomab, as a trifunctional bispecific antibody, co-targets HER2 on tumor cells and CD3 on T cells and mediates ADCC via the Fc fragment. **b** MM-111 specifically targets the HER2/HER3 heterodimer and blocks heregulin (HRG) binding to HER3, and then inhibits HER3 downstream signaling pathways

Ertumaxomab, an intact bispecific antibody, can target HER2 on tumor cells and CD3 on T cells simultaneously, and activate accessory cells via its Fc fragment to exert the function of ADCC. The trifunctional antibody could transiently link immune effector cells to tumor cells and exhibited antitumor activity [35, 36] (Fig. 2a). MM-111 is a novel bispecific antibody, it specifically targets the HER2/HER3 heterodimer and blocks the binding of heregulin (HRG) and HER3, and then inhibits HER3 downstream signaling pathways [37] (Fig. 2b). Moreover, activated T cell armed with HER2-targeted bispecific antibody (HER2Bi-aATC) exhibited significant inhibition in drug-resistant solid tumors [38]. In this review, we summarized the recent advancement of HER2-targeted monoclonal antibodies (trastuzumab, pertuzumab and T-DM1) and bispecific antibodies (MM-111, ertumaxomab and HER2Bi-aATC), especially focus on clinical trial results (completed and ongoing trials of anti-HER2 monoclonal antibodies in Tables 1 and 2, respectively, and clinical trials of HER2-targeted bispecific antibodies in Table 3).

**Table 1:** Completed clinical trials of anti-HER2 monoclonal antibodies

Drug	Identifier	Disease	Phase	Status	Treatment arms	Endpoints
Trastuzumab	NCT01450696 (HELOISE)	HER2+ gastric cancer	3	Completed	a. Capecitabine + cisplatin + trastuzumab (6 mg/kg) b. Capecitabine + cisplatin + trastuzumab (10 mg/kg)	OS a. 12.5 months b. 10.6 months C_trough_ increased in high dose trastuzumab
Trastuzumab	NCT01041404 (ToGA)	HER2+ advanced gastric cancer	3	Completed	a. Trastuzumab + fluoropyrimidine + cisplatin b. Fluoropyrimidine + cisplatin	OS a. 13.8 months b. 11.1 months
Pertuzumab	NCT00545688 (NeoSphere)	HER2+ breast cancer	2	Completed	a. Trastuzumab + docetaxel b. Trastuzumab + docetaxel + pertuzumab c. Trastuzumab + pertuzumab d. Pertuzumab + docetaxel	pCR a. 29.0% b. 45.8% c. 16.8% d. 24.0% PFS a. 81% b. 86% c. 73% d. 73% DFS a. 81% b. 84% c. 80% d. 75%
Pertuzumab	NCT00567190 (CLEOPATRA)	HER2+ metastatic breast cancer	3	Completed	a. Pertuzumab + trastuzumab + docetaxel b. Placebo + trastuzumab + docetaxel	OS a. 56.5 months b. 40.8 months PFS a. 18.7 months b. 12.4 months
Pertuzumab	NCT00976989 (TRYPHAENA)	HER2+ breast cancer	2	Completed	a. Pertuzumab + trastuzumab + FEC b. Pertuzumab + trastuzumab + docetaxel + FEC c. Pertuzumab + trastuzumab + docetaxel + carboplatin	pCR a. 61.6% b. 57.3% c. 66.2% CCR a. 50.7% b. 28.0% c. 40.3%
Pertuzumab T-DM1	NCT00951665	HER2+ locally advanced/metastatic breast cancer	1/2	Completed	a. T-DM1 3.6 mg/kg (Q3W) + paclitaxel 80 mg/m^2^ (QW) b. T-DM1 3.6 mg/kg (Q3W) + paclitaxel 80 mg/m^2^ (QW) + pertuzumab (Q3W)	Total ORR: 50.0% Total CBR: 56.8% Total incidence of grade 3 or worse AEs: 77.3%
Pertuzumab T-DM1	NCT00951665	HER2+ locally advanced/metastatic breast cancer	1/2	Completed	a. T-DM1 3.6 mg/kg (Q3W) + paclitaxel 80 mg/m^2^ (QW) b. T-DM1 3.6 mg/kg (Q3W) + paclitaxel 80 mg/m^2^ (QW) + pertuzumab (Q3W)	Total ORR: 50.0% Total CBR: 56.8% Total incidence of grade 3 or worse AEs: 77.3%
T-DM1	NCT01641939 (GATSBY)	HER2+ advanced gastric cancer	2/3	Completed	a. T-DM1 (2.4 mg/kg) b. Standard taxane therapy	Median follow-up a. 17.5 months b. 15.4 months OS a. 7.9 months b. 8.6 months Incidences of grade 3 or more AEs a. 60% b. 70%
T-DM1	NCT01120184 (MARIANNE)	HER2+ locally advanced/metastatic breast cancer	3	Completed	a. Trastuzumab + taxane b. T-DM1 + pertuzumab c. T-DM1 + placebo	Response rate a. 67.9% b. 59.7% c. 64.2% PFS a. 13.7 months b. 14.1 months c. 15.2 months Incidence of grade 3 or worse AEs a. 54.1% b. 45.4% c. 46.2%
T-DM1	NCT00829166 (EMILIA)	HER2+ locally advanced/metastatic breast cancer	3	Completed	a. T-DM1 b. Capecitabine + lapatinib	ORR a. 43.6% b. 30.8% PFS a. 9.6 months b. 6.4 months OS a. 30.9 months b. 25.1 months Incidence of grade 3 or worse AEs a. 57% b. 41%
T-DM1	NCT01419197 (TH3RESA)	HER2+ breast cancer	3	Completed	a. T-DM1 b. Treatment of physician’s choice	OS a. 22.7 months b. 15.8 months PFS a. 6.2 months b. 3.3 months Incidence of grade 3 or worse AEs a. 40% b. 47%
MM-302	NCT02213744 (HERMIONE)	HER2+ locally advanced/metastatic breast cancer	2/3	Completed	a. MM-302 + trastuzumab b. Chemotherapy of physician’s choice + trastuzumab	OS a. 13.8 months b. 11.1 months

The details of Table 1 derived from http://clinicaltrials.gov/

FEC, 5-fluorouracil, epirubicin, cyclophosphamide, *CCR* clinical complete response, *Q3W* every three weeks, *QW* every week, *CBR* clinical benefit, *rateC*_*trough*_ trastuzumab serum trough concentration, *ORR* objective response rate

**Table 2:** Ongoing clinical trials of anti-HER2 monoclonal antibodies

Drug	Identifier	Disease	Phase	Treatment arms
Trastuzumab	NCT01367002	HER2+ uterine serous cancer	2	a. Carboplatin + paclitaxel + trastuzumab b. Carboplatin + paclitaxel
Trastuzumab	NCT01196390	Esophageal cancer	3	a. Radiation + chemotherapy + trastuzumab b. Radiation + chemotherapy
Trastuzumab	NCT01325207	CNS progression HER2+ breast cancer	1/2	Intravenous trastuzumab
Trastuzumab	NCT02030561	HER2+ breast and gastric cancer	1/2	Trastuzumab + NK cells
Trastuzumab	NCT02598310	HER2+/ER− operable breast cancer	2	Nab-paclitaxel + trastuzumab
Trastuzumab	NCT01340430	HER2+ breast cancer	2	FEC + paclitaxel + trastuzumab
Trastuzumab	NCT01785420	HER2+ operable breast cancer	3	a. Trastuzumab b. Placebo
Trastuzumab	NCT02152943	HR−/HER2+ advanced cancers	1	Everolimus + letrozole + trastuzumab
Trastuzumab	NCT01950182 (SYSUCC-002)	Luminal B2 breast cancer	3	a. Trastuzumab + chemotherapy b. Endocrine therapy + trastuzumab
Trastuzumab	NCT01873833	HER2+ metastatic breast cancer	2	Chemotherapy + lapatinib ditosylate + trastuzumab
Pertuzumab	NCT01996267 (TRAIN-2)	HER2+ breast cancer	3	a. FEC-T + pertuzumab b. PTC + pertuzumab
Pertuzumab	NCT01572038 (PERUSE)	HER2+ breast cancer	3	Pertuzumab + trastuzumab + taxane
Pertuzumab	NCT02229149	HER2+ metastatic breast cancer	2	a. Chemotherapy + trastuzumab + pertuzumab b. Chemotherapy + trastuzumab
Pertuzumab	NCT02896855	HER2+ metastatic breast cancer	3	a. Pertuzumab + trastuzumab + docetaxel b. Placebo + trastuzumab + docetaxel
Pertuzumab	NCT02625441	HER2+ early breast cancer	3	a. Pertuzumab + trastuzumab + docetaxel b. Trastuzumab + docetaxel
Trastuzumab Pertuzumab	NCT02139358	HER2+ metastatic breast cancer	1/2	Gemcitabine + trastuzumab + pertuzumab
Trastuzumab Pertuzumab	NCT01774786	HER2+ gastric or gastroesophageal junction cancer	3	Pertuzumab + trastuzumab + chemotherapy
Trastuzumab Pertuzumab	NCT02536339	CNS progression HER2+ breast cancer	2	Pertuzumab + high-dose trastuzumab
Trastuzumab Pertuzumab	NCT02598427	CNS progression HER2+ breast cancer	1	Intrathecal + pertuzumab + trastuzumab
Trastuzumab Pertuzumab	NCT02581462	HER2+ gastric or gastroesophageal cancer	2/3	a. FLOT b. FLOT + trastuzumab + pertuzumab
Trastuzumab Pertuzumab	NCT02436993	Breast cancer	2	a. Carboplatin + paclitaxel + bevacizumab (HER2−) b. Carboplatin + paclitaxel + trastuzumab + pertuzumab (HER2+)
Trastuzumab Pertuzumab	NCT02411344	HER2+/HR+ breast cancer	2	Pertuzumab + trastuzumab + letrozole
T-DM1 Pertuzumab	NCT02326974	HER2+ breast cancer	2	T-DM1 + pertuzumab
T-DM1	NCT02414646	HER2+ breast cancer	2	T-DM1
T-DM1	NCT02675829	HER2 amplified or mutant cancers	2	T-DM1
T-DM1	NCT01702571	HER2+ locally advanced/metastatic breast cancer	3	T-DM1
T-DM1	NCT02289833	HER2+ locally advanced/metastatic NSCLC	2	T-DM1
T-DM1	NCT01966471	HER2+ primary breast cancer	3	a. T-DM1 + pertuzumab b. Trastuzumab + pertuzumab + taxane
T-DM1	NCT01772472 (KATHERINR)	HER2+ breast cancer	3	a. T-DM1 b. Trastuzumab
MGAH22	NCT01148849	HER2+ cancers	1	MGAH22 (margetuximab)
MGAH22	NCT02492711 (SOPHIA)	HER2+ metastatic breast cancer	3	a. Margetuximab + chemotherapy b. Trastuzumab + chemotherapy
MGAH22	NCT02689284	HER2+ gastric or gastroesophageal junction cancer	1/2	Margetuximab + pembrolizumab
XMT-1522	NCT02952729	HER2+ breast cancer, NSCLC and gastric cancer	1	XMT-1522
DS-8201a	NCT02564900	Advanced solid tumors	1	DS-8201a
SYD985	NCT02277717	Locally advanced/metastatic solid tumors	1	SYD985

The details of Table 2 derived from http://clinicaltrials.gov/

*FLOT* fluorouracil, leucovorin, oxaliplatin, docetaxel, *FEC* fluorouracil, epirubicin, cyclophosphamide, *FEC-T* fluorouracil, epirubicin, cyclophosphamide, trastuzumab, *PTC* paclitaxel, trastuzumab, carboplatin, *CNS* central nervous system, *NSCLC* non-small cell lung cancer

**Table 3:** Clinical trials of HER2-targeted bispecific antibodies

Drug	Targets	Diseases	Treatment arms	Phase	Status	Identifier
Ertumaxomab	HER2/CD3	Metastatic breast cancer	Ertumaxomab	2	Terminated	NCT00452140
Ertumaxomab	HER2/CD3	Metastatic breast cancer	Ertumaxomab	2	Terminated	NCT00522457
Ertumaxomab	HER2/CD3	Her2+ advanced solid tumors	Ertumaxomab	1/2	Terminated	NCT01569412
MM-111	HER2/HER3	Her2+, heregulin+, breast cancer	MM-111	1	Completed	NCT00911898
MM-111	HER2/HER3	Her2+, heregulin+, breast cancer	MM-111 + trastuzumab	1	Completed	NCT01097460
MM-111	HER2/HER3	HER2+ solid tumors	a. Cisplatin + capecitabin + trastuzumab + MM-111 b. Lapatinib ± trastuzumab + MM-111 c. Paclitaxel + trastuzumab + MM-111 d. Lapatinib + trastuzumab + paclitaxel + MM-111 e. Docetaxel + trastuzumab + MM-111	1	Completed	NCT01304784
MM-111	HER2/HER3	HER2+ esophagus cancer, gastroesophageal junction cancer, stomach cancer	a. MM-111 + paclitaxel + trastuzumab b. Paclitaxel + trastuzumab	2	Completed	NCT01774851
HER2Bi-aATC	HER2/CD3	Her2+ neoplasms of digestive system	Interleukin-2 + HER2Bi-aATC	1	Recruiting	NCT02662348
MCLA-128	HER2/HER3	HER2+ malignant solid tumors	a. MCLA-128 dose escalation b. MCLA-128 for breast cancer c. MCLA-128 for ovarian cancer d. MCLA-128 for gastric/GE junction cancer e. MCLA-128 for endometrial cancer f. MCLA-128 for NSCLC	1/2	Recruiting	NCT02912949
GBR1302	HER2/CD3	HER2+ solid tumors	GBR1302	1	Recruiting	NCT02829372
ZW25	Two different epitopes of HER2	HER2+ solid tumors	ZW25	1	Recruiting	NCT02892123

The details of Table 3 derived from http://clinicaltrials.gov/

## Clinical application of anti-HER2 monoclonal antibodies

### Trastuzumab (herceptin)

Trastuzumab is a recombined humanized monoclonal antibody which binds to the extracellular domain IV of HER2 [39]. Trastuzumab is the first anti-HER2 antibody approved by Food and Drug Administration (FDA) in 1998 for the treatment of patients with HER2-overexpressed metastatic breast cancer [40]. Compared with chemotherapy alone, trastuzumab plus chemotherapy exhibited more effective outcomes and better tolerance in HER2-positive breast cancer [41–46]. The combination of aromatase inhibitors (letrozole, anastrozole) and trastuzumab was considered as a treatment option for patients with HER2 positive and hormone receptor positive metastatic breast cancer [47, 48]. 1 year of adjuvant trastuzumab was considered as standard treatment for patients with HER2-positive early breast cancer [49]. A clinical trial (NCT00045032) compared the 2 years of adjuvant trastuzumab versus 1 year of treatment demonstrating that the curative effects of 2 years’ treatment was not more effective than of 1 year’s treatment [50]. Another study did not show that 6 months of adjuvant trastuzumab was non-inferior to 12 months of treatment [51]. In patients with HER2-positive, trastuzumab-refractory metastatic breast cancer, lapatinib in combination with trastuzumab significantly improved progression-free survival (PFS) and clinical benefit rate (CBR) versus lapatinib alone [31]. But the combination of lapatinib and trastuzumab as adjuvant treatment in early HER2-positive breast cancer did not statistically significantly improve disease-free survival (DFS) compared with trastuzumab alone [52]. PAMELA study (NCT01973660) is an open-label, single-group, multicenter, phase 2 trial in patients with early-stage HER2-positive breast cancer treated with trastuzumab and lapatinib. At the time of surgery, 41 of 101 patients (41%) with HER2-enriched subtypes and 5 of 50 patients (10%) with non-HER2-positive subtypes achieved pathological complete response (pCR). The study suggested that HER2-enriched subtype could be supposed as a predictor of pCR before the dual HER2 blockade therapies [53]. The REMAGUS 02 trial compared the pCR, DFS and overall survival (OS) in patients with locally advanced breast cancer treated with neoadjuvant chemotherapy plus celecoxib or trastuzumab. In the HER2-positive population, the combinated application of neoadjuvant and trastuzumab significantly increased pCR rates but not associated with DFS and OS. But axillary pCR may be used as a surrogate predictor of DFS and OS. Patients with HER2-positive breast cancer achieved higher DFS and OS than patients with HER2-negative breast cancer [54]. TRAIN study evaluated the efficacy and toxicity of weekly trastuzumab in combination with paclitaxel plus carboplatin as neoadjuvant treatment in patients with HER2-positive breast cancer. The pCR rate of 108 eligible patients in breast and axilla was 43% (95% CI 33–52) and the 3-year OS was 92% (95% CI 88–98) [55]. Xavier et al. compared the sequential administration of trastuzumab after adjuvant chemotherapy with the concomitant administration of trastuzumab and adjuvant chemotherapy. They found that two regimens achieved similar DFS and OS [56]. Besides breast cancer, in HER2-positive advanced gastric or gastro-esophageal cancer, trastuzumab plus chemotherapy contributed to longer median OS than chemotherapy alone (13.8 months vs 11.1 months) [5].

### Pertuzumab (perjeta)

Like trastuzumab, pertuzumab is a HER2-targeted monoclonal antibody. However, pertuzumab binds to the extracellular subdomain II of HER2 and then inhibited dimerization with other HER receptors, including EGFR, HER3 and HER4 [19]. Hence, pertuzumab has a complementary mode of action compared with trastuzumab. Pertuzumab was first studied in patients with HER2-positive metastatic breast cancer whose disease had progressed during or after previous treatment including trastuzumab [57].

NeoSphere (NCT00545688) is a randomized multicenter, phase 2 study in patients with locally advanced, inflammatory, or early HER2-positive breast cancer. 417 eligible patients were randomly assigned to group A (n = 107, regimen: trastuzumab + docetaxel), group B (n = 107, regimen: pertuzumab + trastuzumab + docetaxel), group C (n = 107; regimen: pertuzumab + trastuzumab), and group D (n = 96; regimen: pertuzumab + docetaxel). The study results demonstrated that the pCR rate in group B (49 of 107 [45.8%]) was higher than in group A (31 of 107 [29.0%]), group C (18 of 107 [16.8%]), and group D (23 of 96 [24.0%]). During the treatment of all regimens, the most common adverse events (AEs) of grade 3 or worse were neutropenia, febrile neutropenia, and leucopenia. The incidence of AEs did not show obvious distinction among groups [58]. The secondary endpoints of NeoSpere study including 5-year progression-free survival (PFS) and DFS were reported by Luca et al. The results support the primary endpoint (pCR) and suggest that pCR could be an indicator of long-term outcome in early-stage HER2-positive breast cancer. Moreover, neoadjuvant pertuzumab plus trastuzumab and docetaxel did not lead to additional and long-term cardiotoxicity. Therefore, the combination of pertuzumab with trastuzumab and docetaxel is beneficial for the improvement of pCR [59]. Directed at NeoSpere study, Giampaolo et al. conducted biomarker analysis. Their results indicated that HER2 positively related with pCR rates and treatment interaction with regimen of pertuzumab, trastuzumab plus docetaxel. But serum transforming growth factor-alpha (TGF-α) showed a negative correlation with pCR rates with pertuzumab plus trastuzumab [59]. Moreover, the analysis of immune modulation of pCR after neoadjuvant HER2-targeting therapies demonstrated that higher expression of PD1, MHC-II and STAT1 were linked with higher pCR, but higher level of PDL1, MHC-I or IF-1 were associated with lower Pcr [60].

The CLEOPATRA study (NCT00567190) is another randomized, double-blind, placebo-controlled phase III trial combining pertuzumab with trastuzumab plus docetaxel in HER2-positive metastatic breast cancer patients [61]. The results of CLEOPATRA trial demonstrated that adding pertuzumab to trastuzumab and docetaxel in patients with HER2-positive metastatic breast cancer significantly prolonged the median OS to 56.5 months. The median OS of placebo group was 40.8 months [62]. The investigator-assessed PFS was 18.7 months in the pertuzumab group, and 12.4 months in the placebo group [63]. The health-related quality-of-life (HRQoL) analysis from CLEOPATRA showed that adding pertuzumab to trastuzumab plus docetaxel had no adverse impacts on overall HRQoL and may prolong the time to deterioration of breast cancer-specific symptoms [64]. The incidence of central nervous system (CNS) metastases as first site of disease progression in patients from CLEOPATRA was similar between arms. Median time to development of CNS metastases as first site of disease progression in pertuzumab arms was longer than placebo arms (15.0 months vs 11.9 months). Median OS was prolonged in pertuzumab arms compared with the placebo (34.4 months vs 26.3 months) [65]. A retrospective analysis of the CLEOPATRA study investigated the prognostic role of tumor-infiltrating lymphocytes (TILs) in advanced HER2-positive breast cancer treated with pertuzumab or placebo in addition to trastuzumab and docetaxel. The results demonstrated that the higher level of stromal TILs was significantly associated with longer OS but not PFS [66]. However, high HER2 protein, high HER2 and HER3 mRNA levels, wild-type PIK3CA, and low serum HER2 extracellular domain (sHER2) were obviously benefit for PFS [67].

In TRYPHAENA study (NCT00976989), 225 patients with HER2-positive breast cancer were recruited and randomized 1:1:1 to receive six neoadjuvant cycles q3w (arm A: 5-fluorouracil, epirubicin, cyclophosphamide [FEC] + trastuzumab + pertuzumab × 3 weeks → docetaxel + trastuzumab + pertuzumab × 3 weeks; arm B: FEC × 3 weeks → docetaxel + trastuzumab + pertuzumab × 3 weeks; arm C: docetaxel + carboplatin + trastuzumab + pertuzumab × 6 weeks). The patients of this study achieved considerable pCR rate in three arms (arm A: 61.6%; arm B: 57.3%; arm C: 66.2%). During treatment, 2 patients (2.7%; arm B) suffered symptomatic left ventricular systolic dysfunction (LVSD) and 11 patients (arm A: 5.6%; arm B: 5.3%; arm C: 3.9%) had declines in LVEF of ≥ 10% points from baseline to < 50% [68]. Based on the TRYPHAENA trial, another study analyzed a panel of biomarkers including HER2, HER3, EGFR, phosphatase and tensin homolog (PTEN), and phosphatidylinositol-4,5-bisphosphate 3-kinase catalytic subunit alpha (PIK3CA) to evaluate the predictive value of these biomarkers. The results demonstrated that HER2 protein and mRNA expression level were corrected/correlated with the pCR rate [69]. A study analyzed the incidence and management of diarrhea in patients with HER2-positive breast cancer during the treatment of pertuzumab-containing through synthesizing three trials including CLEOPATRA (n = 804), NeoSpere (n = 416) and TRYPHAENA (n = 223). The diarrhea was common and manageable adverse event. Besides, diarrhea always occurred during the first treatment cycle of pertuzumab [70].

### Ado-trastuzumab emtansine (T-DM1)

T-DM1 is an antibody–drug conjugate (ADC) targeting HER2 to be approved for the treatment of HER2-positive metastatic breast cancers. It is composed of trastuzumab linked by a stable linker to cytotoxic agent emtansine, an inhibitor of tubulin [71, 72]. DM1 is released in HER2-positive breast cancer cells resulting in cell cycle arrest and apoptosis [73]. Besides, T-DM1 also has the ability of trastuzumab inhibiting HER2-mediated signal pathways [74]. Linker is an important element for T-DM1. The most common linker is valine–citrulline (VC). Study demonstrated that valine–alanine (VA) based antibody–drug conjugates with monomethyl auristatin E is a promising therapy for cancer [75].

A retrospective study analyzed the incidence and time to symptomatic brain progression and median OS in patients with HER2-positive advanced breast cancer treated with single T-DM1 [76]. The median time to brain progression in 16 patients with brain disease before T-DM1 treatment was 9.9 months. The median OS was 15.3 months. In 39 patients without known brain disease before T-DM1, 7 patients (17.9%) developed symptomatic brain progression during treatment with T-DM1 and the time to brain progression was 7.5 months. The median OS was 12.4 months [76]. Studies indicated that T-DM1 combined with paclitaxel or pertuzumab obviously enhanced the antitumor activity [77]. A phase 1b/2a study (NCT00951665) evaluated the maximum tolerated dose (MTD) and feasibility of T-DM1 + paclitaxel ± pertuzumab in HER2-positive locally advanced breast cancer and metastatic breast cancer. The MTD of combination regimen was T-DM1 3.6 mg/kg every 3 weeks or 2.4 mg/kg weekly + paclitaxel 80 ma/m^2^ weekly ± pertuzumab 840 mg loading dose followed by 420 mg every 3 weeks. The Most common severe AEs were neutropenia and peripheral neuropathy that may affect the continuous treatment [78]. EMILIA study (NCT00829166) recruited 991 patients with HER2-positive metastatic breast cancer that previously treated with trastuzumab and taxane. Enrolled patients were randomly assigned to T-DM1 (n = 495) and capecitabine plus lapatinib (control; n = 496). An interim analysis results showed that median PFS assessed by an independent review was 9.6 months with T-DM1 versus 6.4 months with lapatinib plus capecitabine. The objective response rate (ORR) was 43.6% with T-DM1 vs 30.8% with control [79]. A final description analysis indicated that the median OS of T-DM1 group was longer than control group (29.9 months vs 25.9 months). Serious AEs observed in T-DM1 included thrombocytopenia, increased aspartate aminotransferase levels and anemia. The incidence of grade 3 or worse AEs in T-DM1 was lower than in control capecitabine plus lapatinib (48% vs 60%) [80]. GATSBY (NCT01641939) was a randomized, phase 2/3 global study assessing the safety and efficacy of T-DM1 versus taxane treatment in patients with HER2-positive locally advanced gastric or gastro-esophageal junction cancer that progressed during or after first-line therapy. The results suggested that 2.4 mg/kg T-DM1 weekly was the appropriate dose for treatment. However, the T-DM1 group did not show any superiority in median OS compared with the taxane group (7.9 months vs 8.6 months). The incidence of grade 3 or worse AEs in the T-DM1 group was lower than the taxane (60% vs 70%) [81]. TH3RESA study (NCT01419197), a randomized open-label phase 3 trial, compared treatment of T-DM1 with treatment of physician’s choice in patients with previously treated HER2-positive metastatic breast cancer. 602 patients were assigned to T-DM1 group (n = 404) and physician’s choice group (n = 198). The PFS of T-DM1 group was longer than physician’s choice group (6.2 months vs 3.3 months). The incidence of neutropenia, diarrhea, and febrile neutropenia was higher in physician’s choice group than in T-DM1 group. However, thrombocytopenia in T-DM1 group was more common [82]. A final analysis showed that the OS in T-DM1 group was significantly longer than treatment of physician’s choice (22.7 months; 95% CI [19.4–27.5] vs 15.8 months; 95% CI [13.5–18.7]). The incidence of grade 3 or worse AEs was 161 of 403 (40%) patients in T-DM1and 87 of 184 (47%) patients in physician’s choice [83]. However, in MARIANNE study (NCT01120184), 1095 recruited patients with HER2-positive advanced breast cancer were randomly assigned to three groups including trastuzumab + taxane (n = 365), T-DM1 + placebo (n = 367), and T-DM1 + pertuzumab (n = 363). The PFS of three groups did not show significant difference (trastuzumab + taxane: 13.7 months vs T-DM1 + placebo: 14.1 months vs T-DM1 + pertuzumab: 15.2 months). The response rate (67.4% vs 59.7% vs 64.2%) and the incidence of grade 3 or worse AEs (54.1% vs 45.4% vs 46.2%) also had no statistical difference [84].

### Other anti-HER2 monoclonal antibodies

Margetuximab is a Fc-modified anti-HER2 antibody which suppresses the growth of HER2-positive tumor cells and promotes ADCC [85]. A phase 1 study (NCT01148849) was conducted to evaluate MTD, safety and antitumor activity of margetuximab in patients with HER2-overexpressing carcinomas. 66 patients treated with margetuximab including 34 patients received regimen A (intravenous infusion at dose of 0.1–6.0 mg/kg for 3 of every 3 weeks) and 32 patients received regimen B (intravenous infusion at dose of 10–18 mg/kg for once every 3 weeks). 7 of 60 (12%) evaluable patients had partial responses, 31 (52%) had stable disease, and 22 (37%) had progress disease. The MTD was not reached for two regimens. The AEs were mild and manageable. Therefore, margetuximab was a promising single-agent therapy for patients with HER2-positive tumors [85]. 1E11 is an anti-HER2 humanized monoclonal antibody that binds to subdomain IV but distinct from the epitope targeted by trastuzumab. Hence, 1E11 in combination with trastuzumab enhanced the antitumor efficacy in the HER2-overexpressing gastric cancer cell lines [86]. MM-302 is a HER2-targeted liposome encapsulating doxorubicin and single-chain anti-HER2 antibody. The antitumor mechanism of MM-302 is to delivery of doxorubicin and DNA damage [87]. The combination of MM-302 and trastuzumab showed synergistic inhibition for tumor growth in HER2 positive xenograft models of breast and gastric cancer [87]. Moreover, the combination regimen has been studied in clinical trial (NCT02213744) [88]. SYD985 is a novel antibody–drug conjugate composed of trastuzumab and a highly potent DNA-alkylating agent (duocarmycin) [89]. SYD985 induced potent antitumor effects in high HER2 expression (3+) or low expression (2+, 1+) in vitro and in vivo, while T-DM1 only showed high antitumor activity in HER2 3+ tumor cell lines and patient-derived xenograft (PDX) models. This phenomenon has been observed in HER2 positive uterine and ovarian carcinosarcoma [90], epithelial ovarian carcinoma [91], uterine serous carcinoma [92] and breast cancer [93]. DS-8201a contains an anti-HER2 antibody and a potent topoisomerase I inhibitor, exatecan derivative (DX-8951 derivative, DXd). DS-8201a exhibited significant bystander effect due to high drug-to-antibody ratio (DAR) [94]. For the T-DM1-resistant cells (N87-TDMR), DS-8201a remained inhibition for the tumor growth in vitro and in vivo [95]. XMT-1522, another novel ADC, is being studied in preclinical experiment and clinical trials (NCT02952729) [96].

## Preclinical and clinical studies of anti-HER2 bispecific antibodies

### Ertumaxomab

Ertumaxomab, as a trifunctional antibody, eliminates tumor cell lines regardless of HER2 expression level [97]. The ertumaxomab binding epitope on the extracellular of HER2 is different from trastuzumab and pertuzumab. In addition, the antitumor effects of ertumaxomab are mainly depends on the immune-mediated mechanisms. Therefore, for the patients with tumor of low HER2 expression or high HER2 expression but trastuzumab-refractory, ertumaxomab may be a promising therapeutic approach [98]. The trifunctional antibodies induced efficient killing of tumor cells via activating immune effector cells from patients that have received standard chemotherapy and radiotherapy [99].

A phase I clinical trial was conducted to estimate the safety and efficacy of ertumaxomab in patients with metastatic breast cancer. 15 of 17 enrolled patients completed the study with three ascending doses of ertumaxomab (10–200 μg). 100 μg/kg was suggested as the MTD. Severe side-effects including hypotension, respiratory distress syndrome, systemic inflammatory response syndrome, acute renal failure and heart failure were observed in the patients that infused with high doses (150 and 200 μg/kg) ertumaxomab. There were 5 of 15 evaluable patients exhibited antitumor activity (one with complete response, two with partial response and two with stable disease). Besides, cytokines (IL-6, IL-2, TNF-α and IFN-γ) increased in peripheral blood and human anti-mouse/anti-rat antibody was detected in 5 out of 16 evaluable patients [100]. 14 patients with HER2 expressing solid tumors (e.g. breast, gastric, rectal cancer) progressing after standard therapy were enrolled in a phase I clinical trial. Patients were divided into four cohorts and treated with signal ertumaxomab in a weekly escalating dosing regimen. The study results showed that single dose up to 300 μg were well tolerated. Dose limiting toxicity was not detected and the MTD was not reached. All patients experienced treatment-related toxicities. But all AEs were mild and completely reversible. The clinical response to ertumaxomab was observed in 3 of 11 evaluable patients, including one partial remission and two disease stabilizations [101].

### MM-111

MM-111, a novel bispecific antibody consisting of human anti-HER2 and anti-HER3 scFv linked by modified human serum albumin (HSA). HER2 has no known ligands and forms dimerization with other HER members. Ligand-activated HER3 preferentially binds HER2 [102]. HER2 and HER3 play a vital role in transcriptional regulation, proliferation, metastasis and chemo- and radio-resistance [103]. MM-111 simultaneously binding to HER2 and HER3 formed a trimeric inhibitory complex which blocked HER3 and PI3K pathway in the HER2-overexpressing cancers. The combination of MM-111 and trastuzumab or lapatinib potently inhibited growth of HER2-overexpressing tumors in vivo and in vitro [104]. The binding of HER3 and HRG induced HER2:HER3 heterodimer signaling and resistance to trastuzumab in preclinical models [105].

A study demonstrated that HRG reduced the activity of trastuzumab and paclitaxel, but MM-111 remained activity in the presence of HRG in gastric cancer cells. The combination of MM-111, trastuzumab and paclitaxel may be a promising therapy for the patients with HER2 positive gastric cancer [106]. MM-111 and paclitaxel with trastuzumab has been entered into a clinical trial (NCT01774851) in patients with HER2 positive carcinomas of the distal esophagus, gastroesophageal junction and stomach. Besides, treatment with trastuzumab, lapatinib, and MM-111 may be a potent therapeutic approach for the patients with HER2 positive breast cancer [107]. In trastuzumab resistant cells, phosphorylation of EGFR, ERK, CREB, c-Jun, and AFT-1 significantly increased and HER ligands obviously raised at mRNA level. MM-111 and EGFR inhibitors inhibited the growth of trastuzumab resistant cells. Therefore, MM-111 combinates with EGFR inhibitors may be an effective therapeutic regimen for trastuzumab-resistant cancer [108]. MM-111 as a monotherapy or in combination with other HER2-targeted treatment or chemotherapy for HER2 positive cancers was evaluated in serval phase 1 clinical studies (NCT00911898, NCT01097460, NCT01304784).

### HER2Bi-aATCs

HER2Bi-aATCs were generated from human peripheral blood mononuclear cells (PBMC) activated with anti-CD3 monoclonal antibody and interleukin 2 for 14 days and armed with anti-CD3 × anti-Her2 bispecific antibody. In vitro, HER2Bi-aATCs maintained the HER2Bi on the surface and cytotoxic activity as well as the secretion of cytokines and chemokines. PBMC isolated from patients that completed HER2Bi-aATCs infusion showed potent cytolytic activity against breast cancer cell line (SK-BR-3) [109]. Thakur et al. developed an in vitro model which contained naive PBMC, breast cancer cells (SK-BR-3), HER2Bi-aATCs and CpG oligonucleotides (ODNs). In this model, HER2Bi-aATCs targeting and killing tumor cells induced specific antitumor antibody responses [110]. HER2Bi-aATCs exhibited significant cytotoxic activity against HER2 positive colorectal carcinoma cells in vitro. In SCID-Beige mouse model, HER2Bi-aATCs also obviously inhibited the growth of Colo205-luc cells. Compared with unarmed ATCs, HER2Bi-aATCs expressed higher level of activation marker CD69 and secreted more IFN-γ [111]. Amazingly, in HER2 positive melanoma, the same antitumor effects were observed in vivo and in vitro [112]. HER2Bi-aATCs improved the specific cytotoxicity toward PC3 prostate adenocarcinoma cells and increased the secretion of Th1 cytokines (GM-CSF, TNF-α, IFN-γ) compared with unarmed ATCs. In PC3 xenografts, HER2Bi-aATCs also significantly inhibited tumor growth [38]. Due to most glioblastoma simultaneously express EGFR and HER2, bispecific antibody targeting EGFR and HER2 may be an effective strategy for the treatment of glioblastoma. HER2Bi- or EGFRBi-aATCs significantly killed 50–80% primary glioblastoma lines and a temozolomide-resistant variant of U251. Moreover, the increased secretion of Th1 cytokines (IFN-γ, GM-CSF and TNF-α) and Th2 cytokine (IL-13) had been detected [113].

Eight metastatic castrate resistant prostate cancer (CRPC) patients were enrolled into a phase I dose escalation study. 7 of 8 patients were treated with two infusions of HER2Bi-aATCs per week for 4 weeks. Patients who received 40 and 80 × 10^9^ HER2Bi-aATCs doses exhibited 1 partial and 2 minor responses. The prostate specific antigen (PSA) levels significantly decreased in 3 of 7 patients, and the level of IFN-γ and Th1 serum cytokines of two patients had increased. In addition, there were no dose limiting toxicities [114]. Another phase I clinical trial was conducted to determine the safety and efficacy of HER2Bi-aATCs in combination with IL-2 and GM-CSF in 23 patients with advanced breast cancer after enrollment of 14.5 weeks, 13 of 22 (59.1%) evaluable patients achieved a stable disease condition and 9 of 22 (40.9%) had progressive disease. The median OS for all patients was 36.2 months (57.4 months for the HER2 3+ group, 27.4 months for the HER2 0–2+ group). There were no dose limiting toxicities and the MTD was not reached. Encouragingly, HER2Bi-aATCs induced endogenous cytotoxicity and cytokine responses in patients with metastatic breast cancer [115].

### Other anti-HER2 bispecific antibodies

MCLA-128 is a full length IGg1 bispecific antibody targeting HER2 and HER3 [116]. A phase 1/2 dose escalation clinical trial (NCT02912949) to evaluate the safety, tolerability and antitumor activity pf MCLA-128 in patients with solid tumors is ongoing [117]. GBR1302 is a targeting HER2 and CD3 bispecific antibody that has been entered a clinical study (NCT02829372) [118]. ZW25 is a novel humanized bispecific antibody directed against two distinct epitopes of HER2. In high and low HER2 expression, ZW25 showed potent antitumor activity in HER2 positive cancers. A trial (NCT02892123) of ZW25 in patients with advanced HER2-expressing cancers is recruiting patients [119]. Another bispecific antibody MDX-210 co-targeting HER2 and FcγRI, increased the efficacy in vitro when combined with granulocyte-colony stimulating factor (G-CSF) in breast cancer patients overexpressing HER2 [120]. Negrin et al. investigated the ability of ani-HER2 x cancer antigen-125 (CA125) with cytokine-induced killer (CIK) cells against primary ovarian carcinomas. The results suggested that the cytolytic activity of CIK cells with BsAb was significantly higher than CIK cells alone [121]. Due to 12% primary breast cancer expressed both HER2 and CEA, the bispecific antibody simultaneously targeting HER2 and CEA on the same cell obviously enhanced tumor localization [122].

## Conclusions

Trastuzumab has been regarded as a classical drug for the treatment of HER2 positive advanced cancers and showed stronger antitumor efficacy when combined with pertuzumab. The combined use of pertuzumab, trastuzumab and paclitaxel are regarded as standard treatment regimens for patients with HER2 positive metastatic breast cancer. However, drug-resistance to trastuzumab is a key limit factor. HER2-targeted bispecific antibodies including MM-111, ertumaxomab and HER2Bi-aATCs have exhibited significant efficiency for the HER2 positive drug-resistant malignant tumors in preclinical studies. Phase 1 or phase 2 clinical studies demonstrated that bispecific antibodies were safe and feasible for the treatment of HER2 positive cancers. But more studies are required to evaluate the effectiveness of bispecific antibodies. Synergetic application of drugs with different antitumor mechanisms may bring more benefit. Taken together, HER2-targeted immunotherapy including monoclonal antibody and bispecific antibody plays a crucial role in the treatment of HER2 positive cancers.

## Abbreviations

HER2: human epithelial growth factor receptor 2
PI3K: phosphoinositide 3-kinase
Akt: protein kinase B
mTOR: mammalian target of rapamycin
MAPK: mitogen-activated protein kinase
ADCC: antibody-dependent cell-mediated cytotoxicity
T-DM1: ado-trastuzumab emtansine
HRG: heregulin
HER2Bi-aATC: HER2-targeted bispecific antibody armed activated T cell
FDA: Food and Drug Administration
PFS: progression-free survival
CBR: clinical benefit rate
DFS: disease-free survival
pCR: pathological complete response
OS: overall survival
LEVF: left ventricular ejection fraction
AEs: adverse events
PFS: progression-free survival
TGFα: transforming growth factor alpha
HRQoL: health-related quality-of-life
CNS: central nervous system
TILs: tumor-infiltrating lymphocytes
sHER2: serum HER2 extracellular domain
FEC: 5-fluorouracil, epirubicin, cyclophosphamide
LVSD: left ventricular systolic dysfunction
PTEN: phosphatase and tensin homolog
PIK3CA: phosphatidylinositol-4,5-bisphosphate 3-kinase catalytic subunit alpha
ADC: antibody–drug conjugate
VC: valine–citrulline
VA: valine–alanine
MTD: maximum tolerated dose
ORR: objective response rate
PDX: patient-derived xenograft
DAR: drug-to-antibody ratio
HSA: human serum albumin
PBMC: peripheral blood mononuclear cells
ODNs: oligonucleotides
CRPC: castrate resistant prostate cancer
PSA: prostate specific antigen
G-CSF: granulocyte-colony stimulating factor
CA125: cancer antigen-125
CIK: cytokine-induced killer
